# Pancreatic neuroendocrine tumors in patients with tuberous sclerosis: a multicenter study and systematic review

**DOI:** 10.1210/jendso/bvag178

**Published:** 2026-08-05

**Authors:** Kalyan Mansukhbhai Shekhda, John Howat, Yujia Gao, Anat Bel-Ange, Simona Grozinsky-Glasberg, Gregory Kaltsas, Kai Man Alexander Ho, Aimee R Hayes, Dalvinder Mandair, Christos Toumpanakis, Efthimia Karra, Richard A Dixon, Deirdre Donnelly, Elizabeth A Jones, Colin H Jones, Finbar O’Callaghan, Asheesh Sharma, Kirsten Slaney, Aviva Frydman, Rajaventhan Srirajaskanthan, Nabil Kibrya, Martyn Caplin, Frances Elmslie, Ashley Grossman

**Affiliations:** Neuroendocrine Tumour Unit, ENETS Centre of Excellence, Royal Free London NHS Foundation Trust, London NW3 2QG, UK; Department of Clinical Genetics, South West Thames Regional Genetics Services, St George's Hospital, London SW17 0QT, UK; Department of Endocrinology and Diabetes, Addenbrooke's Hospital, Cambridge CB2 0QQ, UK; Neuroendocrine Tumour Unit, ENETS Centre of Excellence, Hebrew University of Jerusalem Faculty of Medicine, Jerusalem 91120, Israel; Neuroendocrine Tumour Unit, ENETS Centre of Excellence, Hebrew University of Jerusalem Faculty of Medicine, Jerusalem 91120, Israel; EKPA-LAIKO, ENETS Centre of Excellence, National and Kapodistrian University of Athens, Athens 115 27, Greece; Neuroendocrine Tumour Unit, ENETS Centre of Excellence, Royal Free London NHS Foundation Trust, London NW3 2QG, UK; Neuroendocrine Tumour Unit, ENETS Centre of Excellence, Royal Free London NHS Foundation Trust, London NW3 2QG, UK; Neuroendocrine Tumour Unit, ENETS Centre of Excellence, Royal Free London NHS Foundation Trust, London NW3 2QG, UK; Neuroendocrine Tumour Unit, ENETS Centre of Excellence, Royal Free London NHS Foundation Trust, London NW3 2QG, UK; Neuroendocrine Tumour Unit, ENETS Centre of Excellence, Royal Free London NHS Foundation Trust, London NW3 2QG, UK; Tuberous Sclerosis Service, Royal United Hospitals Bath NHS Foundation Trust, Bath BA1 3NG, UK; Northern Ireland Regional Genetics Centre, Belfast City Hospital, Belfast, Northern Ireland BT9 7AB, UK; Manchester Centre for Genomic Medicine, Manchester University NHS Foundation Trust, Manchester M13 9WL, UK; Division of Evolution, Infection and Genomics, School of Biological Sciences, Faculty of Biology, Medicine and Health, University of Manchester, Manchester M13 9NT, UK; York & Scarborough Teaching Hospitals NHS Foundation Trust, York YO31 8HE, UK; Tuberous Sclerosis Service, Royal United Hospitals Bath NHS Foundation Trust, Bath BA1 3NG, UK; Great Ormond Street Hospital, London WC1N 3JH, UK; Royal Liverpool Hospital, Liverpool University Hospitals NHS Foundation Trust, Liverpool L7 8XP, UK; Tuberous Sclerosis Service, Royal United Hospitals Bath NHS Foundation Trust, Bath BA1 3NG, UK; Neuroendocrine Tumour Unit, ENETS Centre of Excellence, King's College Hospital NHS Foundation Trust, London SE5 9RS, UK; Neuroendocrine Tumour Unit, ENETS Centre of Excellence, King's College Hospital NHS Foundation Trust, London SE5 9RS, UK; Department of Radiology, King's College Hospital NHS Foundation Trust, London SE5 9RS, UK; Neuroendocrine Tumour Unit, ENETS Centre of Excellence, Royal Free London NHS Foundation Trust, London NW3 2QG, UK; Department of Clinical Genetics, South West Thames Regional Genetics Services, St George's Hospital, London SW17 0QT, UK; Neuroendocrine Tumour Unit, ENETS Centre of Excellence, Royal Free London NHS Foundation Trust, London NW3 2QG, UK

**Keywords:** pancreatic neuroendocrine tumors, tuberous sclerosis complex, insulinoma, TS complex, genetic endocrine syndromes, mTOR inhibitors

## Abstract

**Introduction:**

Pancreatic neuroendocrine tumors (pNETs) are a recognized feature of tuberous sclerosis complex (TSC). The current evidence suggests that pNETs occurring in TSC may exhibit a different clinical course from sporadic cases, but their natural history remains poorly characterized.

**Objective:**

This study aimed to characterize the demographics, clinical presentation, management, and long-term outcomes of TSC-associated-pNETs and to propose possible guidelines for surveillance and management.

**Materials and Methods:**

We conducted a multicenter retrospective review of TSC-pNET patients from 6 UK TSC specialist clinics and from 3 NET referral centers, from 2008 to 2024. Data on demographics, tumor characteristics, management, and outcomes, were collected. A systematic review of the literature from 2009 to 2026 on TSC-pNETs was also performed.

**Results:**

We identified a total of 26 consecutive cases with the TSC-pNET-association in our cohort: 21 cases of pNETs from TSC specialist clinics (1.1% of the population), and 5 cases of TSC-pNETs from the NET referral centers (0.25% of the population). An additional 80 cases were identified from the published literature. We observed a wide spectrum of clinical phenotypes, with the majority being nonfunctioning pNETs (n = 24; 92%), whereas 2 patients were diagnosed with glucagonomas. Surgical intervention was the mainstay initial treatment, the indication being either functional pNETs, or large or symptomatic nonfunctioning pNETs.

**Conclusion:**

TSC-pNETs are rare and mostly nonfunctioning tumors with variable clinical behavior. Due to their uncertain malignant potential, we suggest that baseline pancreatic imaging should be incorporated into TSC surveillance, and we emphasize the need for heightened pNET surveillance and updated management recommendations.

Tuberous sclerosis complex (TSC) is a rare genetic syndrome with an estimated incidence of 1 in 6000 live births (1). It is characterized by the development of typically benign hamartomatous tumors in multiple organs (2). Germline loss-of-function variants in 1 of the 2 implicated genes, *TSC1* or *TSC2*, cause abnormal cellular proliferation and migration due to dysfunction of the TSC protein complex, an inhibitor of the “mechanistic target of rapamycin” (mTOR) pathway (3). Alongside the molecular hallmark, diagnosis is based upon the presence of the typical manifestations, including renal angiomyolipomas, subependymal giant cell astrocytomas, cardiac rhabdomyomas, and classical skin features such as angiofibromas and hypomelanotic macules (4, 5).

Pancreatic neuroendocrine neoplasms comprise mostly pancreatic neuroendocrine tumors (pNETs) and rarely pancreatic neuroendocrine carcinomas (pNECs); pNETs are well-differentiated neuroendocrine tumors that arise from neuroendocrine cells in the pancreas (6). In the general population, they comprise 1% of all pancreatic neoplasms, with an annual incidence of 1 in 100 000 individuals (7). Based on the presence of inappropriate hormone secretion and clinical syndrome, they are classified as functioning or nonfunctioning, the former including insulinomas and glucagonomas (8). Histologically, pNETs are graded as G1 to G3, based on the proliferation index (Ki-67) and cell morphology (9); pNECs are highly aggressive tumors characterized by a high Ki-67 of >20% (usually >55%) together with a poorly differentiated cell morphology (9). Surgical resection is the only curative option for sporadic pNETs (10). However, the indication for surgery in nonfunctioning pNETs is nuanced. It is most appropriate for patients with localized functioning tumors of any size, as well as for nonfunctioning tumors which are ≥2 cm or with a Ki-67 of more than or equal to 3% (G2 and above) and/or if the tumor is growing on sequential imaging. Some authors have proposed a rate of growth of more than 0.5 cm in 6 to 12 months as an indication for surgical resection (11). The smaller than 2 cm well-differentiated G1 nonfunctioning pNETs are considered more indolent and are initially managed with a watch-and-wait surveillance approach (11‐13). There may also be consideration for surgical removal in the presence of metastatic disease in the liver, as excision of ∼90% of tumor mass may improve survival (14‐16).

In approximately 10% of patients, pNETs are associated with genetic conditions such as multiple endocrine neoplasia type 1 (MEN1) (prevalence of pNETs: 25%-80%), MEN4 (very few cases, much less common than MEN1), von Hippel-Lindau (VHL) syndrome (prevalence of pNETs: 12%-17%) and neurofibromatosis type 1 (NF1). Apart from these familial syndromes, they have also been associated with the TSC (17‐21). Although these syndromes share a predisposition to NETs, they considerably differ in their molecular pathogenesis, tumor spectrum, penetrance, and clinical behavior. MEN1-associated pNETs are typically multifocal and have been extensively characterized, whereas VHL-associated tumors generally follow a more indolent clinical course. In contrast, the biological behavior and natural history of TSC-associated pNETs remain poorly defined owing to their rarity and limited available evidence (22).

Unlike in MEN1, where the behavior of pNETs is well described and consensus guidelines have been published (23‐25), the natural history of pNETs in TSC (TSC-pNETs) remains poorly characterized. Despite some evidence suggest that TSC-pNETs differ in histology and presentation compared to sporadic cases, with a lower risk of postoperative recurrence and reduced incidence of metastases, specific guidelines on their management have not been published, with many of the published cases being insulinomas (19, 20, 26). Although the presence of pNETs has been acknowledged in international TSC consensus guidelines, no specific recommendations for surveillance or management have been proposed (4, 5, 27). Recently, the European Standard Clinical Practice Guideline and EXPeRT recommendations for gastro-enteropancreatic neuroendocrine tumors (GEP-NETs) in children and adolescents suggested active surveillance for small (<2 cm), asymptomatic or stable pNETs in patients with hereditary syndromes, including MEN1 and TSC, while parenchyma-sparing surgery should be considered for larger, growing or symptomatic tumors after multidisciplinary team discussion (28). These principles are broadly consistent with current management strategies adopted in adults with small nonfunctioning pNETs (11, 13). However, these recommendations are based primarily on expert consensus, because robust disease-specific evidence for TSC-associated pNETs remains scarce (28).

We have therefore assessed the prevalence, distinctive features, and management of TSC-associated pNETs based experience from 6 UK-based TSC specialist clinics, as well as from 3 major NET referral “*Centres of Excellence*” centers for pNETs in the UK, Greece, and Israel. We have also carried out a systematic review on all published case reports and case series on TSC-pNETs and have summarized the findings. We aimed to characterize their natural history and reports on their management, follow-up, and treatment outcomes, and to propose provisional guidelines for surveillance and management.

## Materials and methods

### For data collection at TSC clinics

We assessed consecutive patients from 6 major TSC specialist clinics across the UK for patients with a confirmed genetic and/or clinical diagnosis of TSC. Patients with TSC and pNETs were identified by searching the internal database *GeneWorks* for patient letters containing the words “*pancreas*” or *“pancreatic*” or after invitation for data collections from various TSC specialist clinics across the UK. All cases were reviewed by a clinical geneticist to confirm a documented diagnosis of a pNET. Of those with a confirmed pNET diagnosis, a panel of characteristic patient data was collected, including sex, age at diagnosis, imaging modality through which the tumor was first detected, and where available grade of pNET, management decision, and follow-up regarding their long-term outcomes.

### For data collection at NET centers

We searched the database of patients who are under the care of a NET referral unit at 3 ENETS *Centers of Excellence* from 2008 to 2024: UK (Royal Free Hospital, London), Israel (Hadassah Medical Organisation, Jerusalem), and Greece (National and Kapodistrian University of Athens), with a histologically proven diagnosis of pNET and clinical and/or genetic diagnosis of TSC. At all these centers, patients with pNETs are followed up regularly with cross-sectional imaging computed tomography/magnetic resonance imaging (CT/MRI) every 6 to 12 months and/or biochemical analysis with fasting gut hormone profile, according to current ENETS guidelines (11). However, there is currently no specific protocol for patients with a concomitant diagnosis of TSC and pNETs.

Since this study was a retrospective audit of service, this was registered with hospital audit department; therefore, ethical approval was not required under the UK Policy Framework for Health and Social Care Practice (Audit Registration numbers: Royal Free Hospital: RFHBU_89224/25, St George's University NHS Foundation trust:AUDI004849).

### Method for the systematic review of the published literature

**Figure 1:**
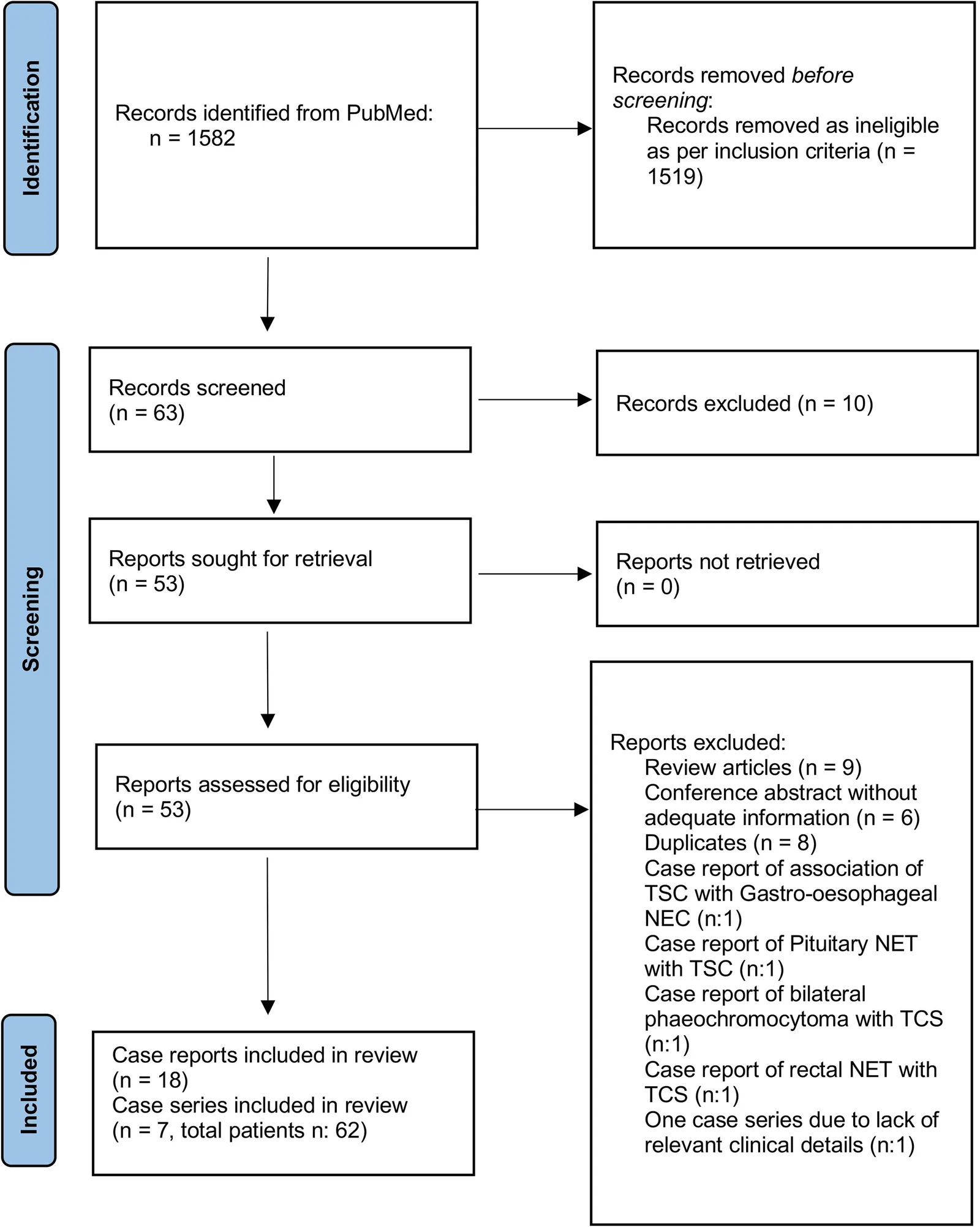
PRISMA flow diagram.

The inclusion criteria for this systematic review encompassed any published case reports, case series, or other studies evaluating concomitant diagnoses of TSC and NETs. Two authors (K.M.S. and Y.G.) performed an independent literature search in PubMed terminating on July 25, 2026 using the following terms: (“*tuberous sclerosis*” OR “*tuberous sclerosis complex*” OR “*TSC*” OR “*TS*”) AND (“*neuroendocrine tumors*” OR “*NET*” OR “*Pancreatic neuroendocrine tumours*” OR “*PanNET*”). We restricted the years from 2009 to 2026, as literature prior to 2009 had already been reviewed systematically and published (17). Additionally, when multiple publications reported overlapping patient cohorts from the same institution, only one dataset was included in the pooled analysis to avoid duplicate patient inclusion. The search retrieved 1582 articles. All abstracts were screened to assess for eligibility. Of these, 63 papers underwent full-text review after duplication removal and excluding articles. The references of all papers were also screened to identify additional relevant studies. Finally, 18 case reports and 7 case series were included for the data collection. A Preferred Reporting Items for Systematic Reviews and Meta-Analyses (PRISMA) flow diagram for the method is described in Fig. 1, in accordance with PRISMA guidelines (29).

### Statistical analysis

Given the nature of this study, we employed descriptive statistics to report demographics, clinical, laboratory, and radiological investigations. Means and SD were calculated for continuous variables, and frequencies and percentages were used for dichotomous variables. Microsoft Excel was used for the data collection and analysis.

## Results

### Results of the UK and European patient cohort

#### Demographics

In total, 26 cases of TSC-pNETs were identified: 21 from 6 UK tuberous sclerosis complex specialist clinics (Table 1) and 5 additional cases from 3 NET referral centers: 2 from the UK, 2 from Greece, and 1 from Israel (Table 2). The median age at diagnosis was 37 years (range 13-69). Of these cases, 11 patients were male and 15 were female.

**Table 1:** Summary of the clinical information relating to cases from tuberous sclerosis clinics in the UK

Patient	Age at Dx and sex	Year pNET detected	Imaging indication	Imaging modality	pNET grade	Functioning	Management	Outcome
**1**	13 F	2023	Surveillance	MRI	Grade 1	No	Surgical resection	No recurrence (1 year)
**2**	19 M	2020	Surveillance	MRI	Grade 1	No	mTOR inhibitor started	Tumor has shrunk from 3.7 to 3 cm after 4 years
**3**	19 F	2024	Surveillance	MRI	Grade 1	No	Watch and wait	Tumor stable
**4**	22 M	2015	Surveillance	MRI	Grade 1	No	Surgical resection	No recurrence (9 years)
**5**	25 F	n/a	Incidental	CT	n/a	No	Watch and wait	Tumor stable
**6**	26 M	2008	Surveillance	USS	Grade 1	No	Surgical resection	No recurrence (15 years)
**7**	27 F	2010	Surveillance	USS	Grade 2	Glucagonoma	Surgical resection	Recurrence detected 1-year post-resection. Patient died from complications of recurrence 10 years after detection
**8**	28 F	2012	Surveillance	MRI	Grade 1	No	Surgical resection	No recurrence (12 years)
**9**	29 M	2017	Surveillance	MRI	Grade 2	No	Watch and wait	Developed metastatic disease 6 years post-identification
**10**	30 F	2023	Surveillance	MRI	Grade 1	No	Watch and wait	Tumor stable
**11**	31 M	2017	Incidental	CT	Grade 1	No	Watch and wait	Tumor stable
**12**	33 M	2012	Surveillance	CT	Grade 1	No	mTOR inhibitor started	Tumor stable
**13**	35 F	2002	Symptomatic	CT	Grade 1	Glucagonoma	Surgical resection	Local recurrence detected 17 years post-resection Treated with IRE, stable 6 years post-recurrence
**14**	37 F	2024	Surveillance	MRI	Grade 1	No	Planned surgical resection	Surgery planned
**15**	38 F	2024	Surveillance	MRI	Grade 1	No	Somatostatin analogue started	Tumor stable
**16**	40 F	n/a	Surveillance	CT	n/a	No	Watch and wait	Tumor stable
**17**	46 F	2016	Incidental	CT	Grade 2 with hepatic metastasis	No	Surgical resection and somatostatin analogue followed by RLT	Recurrence of hepatic metastases 1-year post-resection
**18**	53 F	2023	Surveillance	USS	Grade 2	No	Surgical resection	No recurrence (1 year)
**19**	67 M	2017	Surveillance	USS	Grade 1	No	Surgical resection	Hepatic metastases detected 4 years post-resection
**20**	69 M	2021	Surveillance	CT	n/a	No	Watch and wait	Tumor stable
**21**	42 F	2022	Surveillance	MRI	Grade 3	No	Everolimus started	Progression of metastatic disease

Lesion size was not available to include in the data set.

Abbreviations: CT, computed tomography; MRI, magnetic resonance imaging; pNET, pancreatic neuroendocrine tumor; RLT, radioligand therapy; USS, ultrasound scan.

**Table 2:** Summary of the clinical information relating to cases reported in 3 NET units in the UK, Greece, and Israel

Patient	Age at pNET Dx and sex	Year pNET detected	Imaging indication	Imaging modality	Size	pNET grade	Functional imaging	Metastases	Functioning	Management	Outcome
**1 (Israel NET centre)**	32 F	2023	Surveillance	MRI	10.5 cm	Grade 2	^68^Ga-DOTATATE scan: avid pancreatic mass FDG PET: N/A	No	No	Surgical resection	No recurrence (2 years)
**2 (Greece NET centre)**	40 F	2022	Surveillance	MRI	5.8 cm	Grade 2	^68^Ga- DOTATATE scan: N/A ^18^FDG PET scan: nonavid pancreatic mass	No	No	Surgical resection	No recurrence (2 years)
**3 (Greece NET center)**	32 F	n/a	Surveillance	CT	N/A	Grade 1	^68^Ga-DOTATATE scan: N/A FDG PET scan: N/A	No	No	Everolimus started	Died due to renal and liver dysfunction unrelated to NET
**4 (UK NET center)**	54 M	2010	Symptomatic	CT	5.6 cm	Grade 2	^68^Ga- DOTATATE scan: N/A FDG PET scan: N/A Octreotide scan: avid pancreatic lesion with 4 liver metastases	Liver metastases	No	Somatostatin analogue started for progression of his metastatic disease	Died 6 years post diagnosis (cause unclear)
**5 (UK NET center)**	43 M	2016	Surveillance	MRI	1.8 cm	Grade 1	^68^Ga- DOTATATE scan: avid pancreatic mass. ^18^FDG PET scan: N/A	No	No	Surgical resection	No recurrence (3 years)

Abbreviations: CT, computed tomography; MRI, magnetic resonance imaging; PET, positron emission tomography; pNET, pancreatic neuroendocrine tumor.

Of 1890 patients with a confirmed diagnosis of TSC under regular follow-up in the TSC centers, 21 patients were found to have a pNET, a prevalence of 1.1% in this cohort. Of 1997 patients with pNETs from NET referral centers (UK: 865 patients; Israel: 400 patients; and Greece: 732 patients), we respectively identified 2, 1, and 2 patients who had a confirmed diagnosis of TSC, an overall prevalence of 0.25%. In this cohort of patients with pNETs, a total of 210 patients had MEN1 syndrome, and 58 patients had VHL syndrome.

#### Diagnosis

Of the 26 patients with a TSC-pNET, 21 were detected following routine surveillance abdominal imaging. A further patient had imaging due to the presence of clinical symptoms of a glucagonoma, while 4 were detected as incidental findings—1 in a patient who was not diagnosed with TSC at the time and had imaging following an out-of-hospital cardiac arrest, and 3 who presented to hospital with abdominal pain. Regarding primary imaging modalities, 13 were detected on MRI, 9 through CT, and 4 using ultrasonography (USS). The size of tumors in patients from the NET centers varied from 1.8 cm to 10.5 cm.

In 23 patients the grade was available: 16 pNETs were grade 1, while 6 were grade 2, and 1 was grade 3. In 3 cases, pNETs were diagnosed based on imaging findings but no histology reports were available. Of 5 patients from NET centers, the results of functional imaging were available in 4 patients. These are described in Table 2. Functional imaging was not available for the TSC centers.

Of the total of 26 patients, one of the grade 2 tumors showed hepatic metastases at presentation, while 5 later became metastatic; 24 were nonfunctioning, one had symptomatic histologically confirmed glucagonoma, and one was asymptomatic with histology indicating a glucagonoma.

#### Management and outcomes

**Figure 2:**
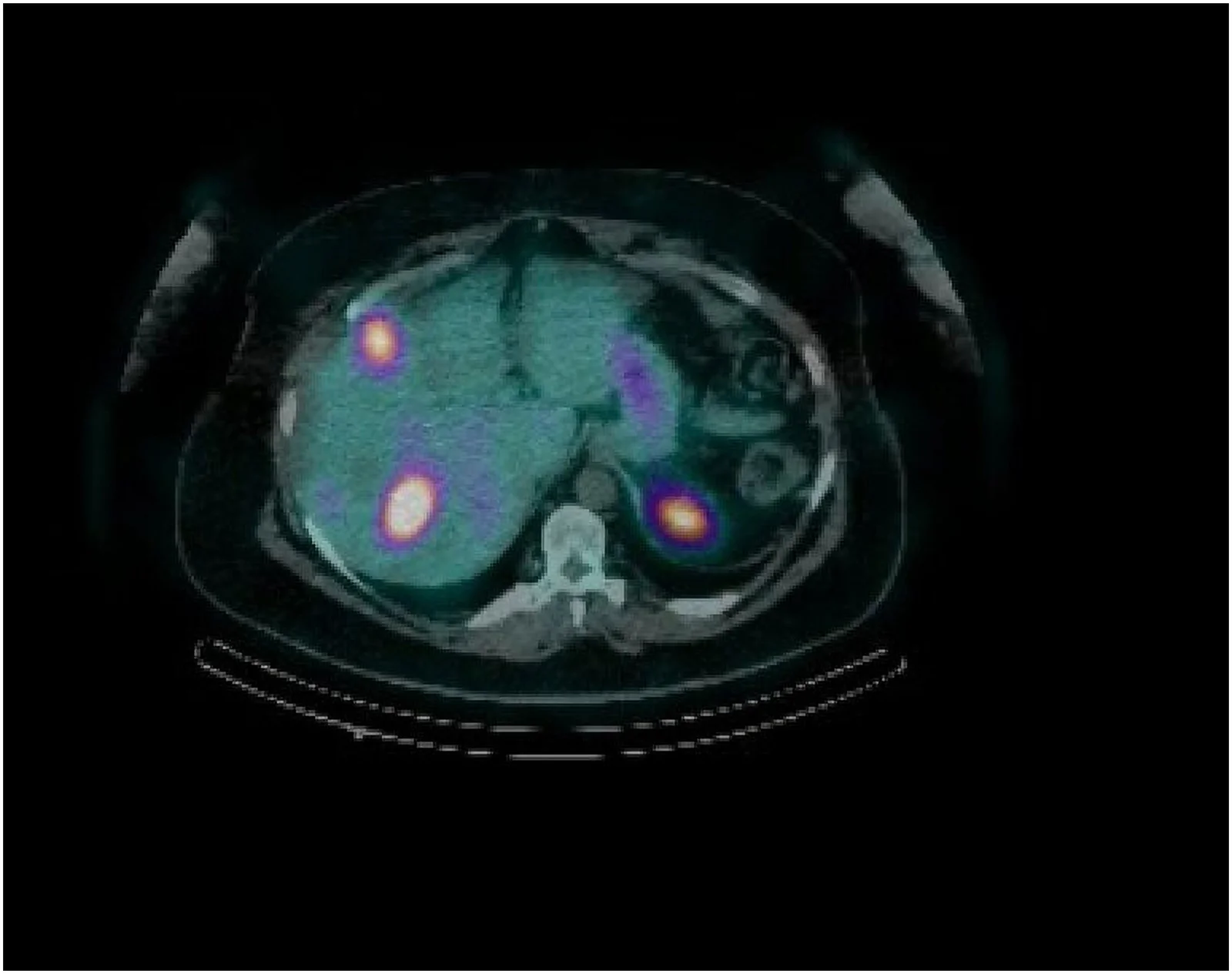
Cross-sectional image of a patient showing multiple ^68^Ga-DOTATATE-avid liver metastases in a patient with TSC and a metastatic pNET.

Around half of the patients (n = 12, 46%) underwent surgical resection; 6 patients received medical therapy with either mTOR inhibitors or somatostatin analogues, while 7 were managed with active surveillance. One additional patient was initiated on a somatostatin analogue with a planned surgical resection following a recent diagnosis. Of the 14 cases where nonsurgical management was planned, 10 pNETs have remained stable, including 1 in whom mTOR inhibitor therapy led to a 19% reduction in pNET size after 4 years. One conservatively managed grade 2 pNET evolved into inoperable metastatic disease 6 years after initial diagnosis. The patient with a grade 3 pNET also had progressive metastatic disease and received everolimus on progression of the disease. Two patients with medically managed pNETs have died, but in both cases the pNETs were unlikely to have contributed to their death. One patient (Patient 17) with a grade 2 pNET developed metastatic disease 1 year after surgical resection and received radioligand therapy (RLT) with ^177^Lu-DOTATATE, showing a partial response to the treatment. Figure 2 depicts ^68^Ga-DOTATATE-avid multiple liver metastases in patient 17.

Of the 12 patients who underwent surgical resection, 8 have shown no postoperative recurrence or metastases. Postoperative recurrence was seen in 4 cases (Grade 1: 2, Grade 2: 2), 2 of whom had glucagonomas. One of these patients died from complications of pNET recurrence 11 years after initial detection.

Details of the characteristics of cases based on the center of presentation (TSC specialist clinic vs NET referral center) are described in Tables 1-3.

**Table 3:** Summary of information and characteristics of cases based on center of presentation

	TSC centers	NET centers	Overall
**Mean age at the diagnosis**	34 years (13-69 years)	45 years (32-57 years)	37 years (13-69 years)
**Sex**	Female = 13	Female = 2	Female = 15
	Male = 8	Male = 3	Male = 11
**Imaging indication**	Surveillance = 17	Surveillance = 4	Surveillance = 21
	Incidental = 3	Incidental = 0	Incidental = 3
	Symptomatic = 1	Symptomatic = 1	Symptomatic = 2
**Imaging modality**	MRI = 10	MRI = 3	MRI = 13
	USS = 4	USS = 0	USS = 4
	CT = 7	CT = 2	CT = 9
**pNETs grade**	Grade 1 = 13	Grade 1 = 3	Grade 1 = 16
	Grade 2 = 4	Grade 2 = 2	Grade 2 = 6
	Grade 3 = 1	Grade 3 = 0	Grade 3 = 1
	No biopsy = 3	No biopsy = 0	No biopsy = 3
**Functionality**	Functioning = 2 (glucagonoma)	Functioning = 0	Functioning = 2
	Nonfunctioning = 19	Nonfunctioning = 5	Nonfunctioning = 24
**Management**	Surgical resection = 9	Surgical resection = 3	Surgical resection = 12
	Wait and watch = 7	Wait and watch = 0	Wait and watch = 7
	mTOR inhibitor = 3	mTOR inhibitors = 1	mTOR inhibitor = 4
	Surgical resection and somatostatin analogues = 1	Surgical resection and somatostatin analogues = 0	Surgical resection and somatostatin analogues = 1
	Somatostatin analogues = 1	somatostatin analogues = 1	Somatostatin analogues = 2
**Outcome**	Cured = 5	Cured = 3	Cured = 8
	Stable disease = 10	Stable disease = 0	Stable disease = 10
	Died = 1	Died = 2	Died = 3
	Progression of metastatic disease = 1	Recurrence or metastases = 0	Recurrence or metastases = 5
	Recurrence or metastases = 4		

Abbreviations: CT, computed tomography; MRI, magnetic resonance imaging; mTOR inhibitor, mechanistic target of rapamycin; NET, neuroendocrine tumor; pNETs, pancreatic neuroendocrine tumors; TSC, tuberous sclerosis complex; USS, ultrasound scan.

### Results of the systematic review of the studies published until July 2026 (described in Tables 4 and 5)

#### Demographics

A total of 80 cases (case reports n = 18, case series n = 62 patients in 6 studies) were reported in PubMed from January 2009 to July 2026. The majority of published reports were from the USA (n = 53) followed by Europe (n = 8). The association was slightly more prevalent in male (n = 45) compared to female (n = 35) individuals. Of the 18 case reports, the median age at the diagnosis of TSC was 29 years (range, 0.25-47) which was slightly less compared to the median age of the diagnosis of the pNET (31 years [range, 3.5-67 years]). Only 1 patient had only a clinical diagnosis of TSC with negative genetic testing (42). Details of the other findings are shown in Tables 3 and 4.

**Table 4:** Overview of cases reported in literature from 2009 to 2026

Country and year	Age (years)	Sex	TSC diagnosis age (years)	Genetic diagnosis (mutations in *TSC1* or *TSC2* gene)	NET diagnosis age (years)	Type of NET	Treatment of NET	Follow-up and outcome	Remark, additional information
USA 2009 (30)	24	Male	n/a	n/a	24	Well-differentiated nonfunctioning pNET	Surgical removal of pNET	Cured at the time of publication	pNET was around 8 cm in size
USA 2012 (31)	15	Male	15	TSC2	15	Well-differentiated nonfunctioning pNET	Surgical removal of pNET	n/a	pNET was presenting feature for TSC
Spain 2012 (32)	31	Male	n/a	n/a	31	Well-differentiated nonfunctioning pNET	Distal pancreatectomy	Cured at the time of publication	n/a
Italy 2013 (33)	10	Male	4 months	TSC2	10	Well-differentiated nonfunctioning grade 2 (Ki-67 of 4%) pNET	Distal pancreatectomy	Cured at the time of publication	Size of pNET is not mentioned
New Zealand 2017 (34)	23	Male	n/a	n/a	23	2 Well-differentiated pNET, Grade 1 One of them insulinoma but no staining for insulin performed	Distal pancreatectomy	Cured at the time of publication	Patient presented with seizures, hypoglycemia Biochemical confirmation of hyperinsulinemic hypoglycemia Tail of pancreas lesion 3.5 × 2.6 × 2.9 cm but intraoperatively another small <1 cm size lesion found
Germany 2017 (35)	41	Male	n/a	TSC2	41	Well-differentiated nonfunctioning pNET grade 2: Ki67 5%	Surgery	Patient developed metastases 12 months after surgery Successfully treated with mTOR inhibitors	Authors did immunohistochemistry staining for mTOR pathway and patient was found to have strong activation of the Akt/mTOR pathway. pathway. Subsequently, authors decided to treat patient with everolimus. Patient exhibited partial response (6% reduction of liver tumor burden with 2 lesions even no longer visible)
USA 2018 (36)	35	Female	n/a	TSC1	35	Grade 1 nonfunctioning well-differentiated pNET	Surgery	Cured at the time of publication	n/a
London 2018 (37)	67	Female	47	n/a	67	Well-differentiated grade 1 insulinoma	Surgery	Cured at the time of publication	Patient had history of CKD Authors used ^68^Ga-DOTATATE to confirm the diagnosis of pNET
USA 2019 (38)	3.5	Male	3 months	TSC1	3.5	Well-differentiated nonfunctioning pNET, grade 2, Ki-67 of 15%	Enucleation of pancreatic mass	Cured at the time of publication	Parents were negative for TSC genetic mutation Tumor cells were focally positive for glucagon but negative for insulin, somatostatin, gastrin
India 2019 (39)	17	Female	n/a	n/a	17	Well-differentiated nonfunctioning pNET of grade 1: Ki-67 of 2%	Whipple pancreatoduodenectomy	Cured at the time of publication	n/a
Brazil 2020 (40)	45	Male	45	TSC2	45	Well-differentiated low-grade pNET	n/a	n/a	Patient was diagnosed to have TSC and pNET at the same time. Family history of CNS tumors in father and brother
Saudi Arabia 2021 (41)	47	Male	n/a	n/a	47	Well-differentiated low-grade (Ki-67 of <2%) pNET, insulinoma	Enucleation	Cured at the time of publication	Insulinoma was detected incidentally but retrospectively patient had symptoms of hypoglycemia prior to the diagnosis
Georgia 2021 (42)	34	Female	33	Clinical diagnosis Genetic test negative	34	Pancreatic NET but no histological confirmation	n/a	n/a	Patient had diagnosis of pancreatic cyst at the time of suspicion of clinical diagnosis of TSC Patient was heterozygous to COL4A4 gene
Netherlands 2021 (43)	11	Male	n/a	n/a	11	Nonfunctioning pNET	Robotic distal pancreatoctomy	n/a	This case is predominantly video abstract for surgical technique for distal pancreateoctomy in a young patient
China 2022 (44)	43	Male	43	TSC2	43	pNET	Surgical removal of pNET	n/a	pNET is the presenting feature of TSC
Turkey 2022 (45)	32	Male	27	n/a	32	Functioning insulinoma	Surgical removal of the distal pNET	Cured at the time of publication	n/a
USA (46)	31	Male	31	TSC1	31	Well-differentiated nonfunctioning pNET	Distal pancreatectomy and splenectomy	Cured at the time of publication	n/a
Overall: USA: 5 Saudi Arabia: 1 Brazil: 1 India: 1 Europe: 6 Turkey: 1 China: 1 UK: 1 New Zealand: 1	median age: 31 (3.5-67 years)	Male: 13 Female: 5	Median age TSC diagnosis: 29 (0.25-47)(for 7 patients) n/a: 8 patients	TSC1: 3 TSC2: 6 Clinical diagnosis: 1 n/a: 8	median age of NET diagnosis: 31 (3.5-67 years)	Well-differentiated pNET: 13 Insulinoma: 5	Enucleation: 2 Surgery: 13 Surgery followed by mTOR inhibitor: 1 Data n/a: 2	Data n/a: 5 Cured at the time of publication: 12 Metastases 12 months after surgery: 1	

Abbreviations: CNS, central nervous system; NET, neuroendocrine tumor; pNET, pancreatic neuroendocrine tumor; TSC, tuberous sclerosis complex.

**Table 5:** Overview of published case series from 2009-2026

	Country, year	Number of cases	Sex	Mean TSC diagnosis age	Average/median age at NET diagnosis	Type of NET	Treatment of NET	Follow-up and outcome	Remarks and additional information	Reference
1	USA 2021	16	9 males 7 females	n/a	15 years (range 3-55 years, IQR: 15.5-25)	pNET	Surgery: 6 mTOR inhibitors: 5 Surgery and mTOR inhibitors: 3 No treatment: 2	No long-term follow-up data available	Authors had monitored change of the size of pNET Tumor growth rate was slightly less in patients who had treatment with mTOR inhibitors	Mowrey et al (19)
2	USA 2022	13	7 males 6 females	n/a	n/a	Grade 1 pNET: 6 Grade 2 pNET: 4 No grading available: 3 All nonfunctioning	Surgery in all patients	12 patients no recurrence at the time of publication 1 patient had recurrence 33.8 months after surgery	Authors have reviewed outcome of surgical resection of pNETs in TSC patients	Evans et al (18)
3	Multicenter study 2021	12	5 males 7 females	n/a	21.11 years (9-63 years)	n/a	8 patients received treatment	n/a	The study is from TOSCA. One patient had duodenal NET and one patient had pituitary adenoma	Sauter et al (47)
4	USA 2017	2 confirmed pNET	2 males	n/a	5 and 12 years of age	2 nonfunctioning pNET	1 surgical removal 1 had surgical removal and everolimus therapy	Patient who had everolimus therapy had reduction in the tumor burden post-treatment	Authors reviewed centralized data of 150 patients with TSC, included 55 patients who had serial MRI imaging for surveillance	Koc et al (48)
5	USA 2012	6	3 males 3 females	n/a	31.1 years (10-51 years)	5 grade 1 pNET 1 grade 2 pNET	n/a	n/a	Authors have studied cases of TSC who were found to have pancreatic mass/cysts	Larson et al (49)
6	USA 2025	11	6 males 5 females	2 (0-13) years	13 (8-22) years	n/a	Surgery: 4 No treatment: 7	n/a	n/a	Li R et al (50)
7	Greece 2025	pNETs: 2	Male: 0 Female: 2	39 years, 43 years	n/a	Grade n/a: 1 Grade 2: 1	Surgery: 1 No treatment: 1	n/a	n/a	Spanou M et al (51)
	Total:	pNET: 62	Male: 32 Female: 30	n/a	—	Nonfunctioning pNET: 51	Surgery only: 25 mTOR inhibitors only: 5 Surgery and mTOR inhibitors: 4 No treatment: 10 Information n/a: 18	Majority data n/a One patient had recurrence after 33 months of surgery Rest of the patient didn’t have recurrence		

Abbreviations: IQR, interquartile range; mTOR, mechanistic target of rapamycin; n/a, not available; NET, neuroendocrine tumor; pNET, pancreatic neuroendocrine tumor; TOSCA, Tuberous Sclerosis to increase disease awareness; TSC, tuberous sclerosis complex.

#### Diagnosis

Regarding the case reports, the majority of the patients presented following symptoms of either tumor-related abdominal discomfort or of insulin hypersecretion due to an insulinoma. Of 80 patients, 5 had a diagnosis of an insulinoma, while the remaining had well-differentiated nonfunctioning pNETs. In the majority of the 80 patients, 62/80, all from case series, the diagnosis was made based on routine surveillance abdominal imaging.

#### Management and outcomes

The majority of the patients had surgical intervention for their well-differentiated pNET or an insulinoma. Of the total, 40 had surgical removal of the pNET, 5 had both surgery and mTOR inhibitors, 5 had mTOR inhibitors alone, while 10 had no treatment. Information regarding the treatment received was not available for 20 patients. Most of the patients were disease-free at the time of publication, except for 2 patients who had developed metastatic disease 12 and 33 months after surgery, but no further information was given (18, 35).

## Discussion

Although the prevalence of pNETs in patients with TSC remains poorly characterized, with an estimated range from 0.65% to 9% in this population (19, 48), it represents an increasingly recognized extra-renal manifestation of TSC. In our retrospective analysis including data from both TSC centers as well as those specializing in NETs, we have reported on 26 TSC patients who were found to have pNETs, and we have conducted a systematic review of the literature that described a further 80 patients. In a recently published study, the prevalence of pNETs in TSC patients was found to be 1.7% (n = 2/115), which is closely aligned with our prevalence of 1.1% (51). Collectively, our findings highlight that although pNETs are uncommon but potential extra-renal manifestation of TSC, they may present at a younger age, exhibit variable clinical behavior, and especially in view of their molecular derangement, they may be suitable for treatment with mTOR inhibitors such as everolimus. The median age at diagnosis of TSC-pNETs in our cohort was 34 years (range, 13-69 years), and 31 years (range, 3.5-67 years) in previously reported cases. Compared with sporadic pNETs, which are more prevalent in the sixth decade of life, TSC-associated tumors frequently present during childhood or early adulthood, highlighting the importance of maintaining a high index of suspicion in young patients with TSC (28, 50, 52). Early diagnosis is facilitated by routine surveillance imaging, allowing many lesions to be detected before symptoms develop and enabling timely multidisciplinary management (28, 50, 53). Additionally, young patients presenting with pNETs should be assessed for an underlying hereditary tumor predisposition syndrome. Genetic counseling and germline testing for MEN1, VHL, NF1, and TSC should be guided by the clinical phenotype and family history (28). Of the 26 patients in our cohort, 24 patients had well-differentiated, nonfunctioning pNETs while 2 patients had functioning pNETs with glucagon hypersecretion or immunostaining. Similarly, in previously published reports, where most patients had well-differentiated, nonfunctioning pNETs, 5 patients had insulinomas. While the incidence of insulinoma with TSC is well documented, our 2 incidences of glucagonoma in our cohort are to our knowledge the first to be reported (17, 34, 37, 45, 54, 41).

The *Tuberous Sclerosis Complex 1* (*TSC1*) gene and *Tuberous Sclerosis Complex 2* (*TSC2*) encode proteins hamartin and tuberin, respectively (17). In the normal population, these proteins form a complex that inhibits the mTOR signaling pathway, a crucial regulator of cell growth and proliferation (55). In patients with TSC, loss-of-function mutations in the *TSC1* and/or *TSC2* gene and activation of mTOR leads to activation of uncontrolled cell growth and proliferation in many tissues. This mechanism leads to various manifestations of TSC, such as renal angiomyolipomas and subependymal tubers. The role of the mTOR signaling pathway has also been demonstrated to be occasionally involved in tumorigenesis and the progression of sporadic NETs, with 4% showing somatic mutations of *TSC1* or *TSC2*, but a larger proportion showing copy number changes (57). Additionally, everolimus is an established therapeutic target in NETs (57). In a recent study from Brazil, the authors screened 93 nonsyndromic patients with GEP-NETs of any grade for germline mutations of *MEN1*, *RET*, *VHL*, *NF1*, *TSC1*, and *TSC2,* and found 8 patients with *TSC2* germline missense mutations (58). Despite this evidence, it remains unclear as to whether pNETs in TSC constitute a clinico-pathological entity or whether they represent an increased predisposition to neoplasia within this genetic background. Regardless of our advanced understanding of the pathophysiology of TSC and NETs and an increasing body of evidence to suggest possible association of pNETs with TSC, international guidelines currently do not specifically mention screening of TSC patients for pNETs (5). Apart from pNETs, there are reports of other neuroendocrine tumors, such as bilateral pheochromocytoma and ganglioneuroma (59), metastatic neuroendocrine carcinoma of the gastro-esophageal junction (60), a nonfunctioning pituitary tumor with bilateral cavernous sinus invasion (61), a well-differentiated L-cell neuroendocrine tumor with low proliferative features of the rectum (62), and Cushing disease (63), associated with TSC, which could potentially have the same pathophysiological pathway for the disease development as pNETs.

From a clinical standpoint, recognizing pNETs as a manifestation of TSC has its implications for surveillance strategies. Current international guidelines focus on renal, neurological, and pulmonary manifestations of TSC, but pancreatic involvement has received limited attention (1, 4, 5, 19). While the majority of such pNETs are slow-growing, low-grade, well-differentiated pNETs, there are reports of some having developed metastases, and this risk is likely higher in the grade 2 and grade 3 pNETs in our limited data set. From the point of view of centers specializing in pNETs and other neuroendocrine tumors, the relative rarity of pNETs and usual association with other clinical manifestations of TSC suggests that specific germline screening for TSC is not generally indicated routinely in patients with pNETs unless other associated features of TSC are present, despite the report of Asprino et al (58).

In TSC centers, the prevalence of pNETs was around 1%. When diagnosed, these patients were followed up regularly. In our cohort as a whole, 13 cases were managed conservatively, and 9 of these tumors have remained stable. There have been 2 deaths, probably unrelated to the pNETs. One case, which evolved into metastatic disease, was initially misdiagnosed as a pancreatic pseudocyst and only identified as a pNET following the development of metastases. The patient with the grade 3 pNET presented with metastatic disease at the time of diagnosis. Given such heterogeneity, it is prudent to propose that initial screening for pNETs should be specifically requested alongside surveillance for TSC-related renal disease. Additionally, if during surveillance scans any suspicious pancreatic mass is found, patients should be investigated for a possible pNET, with functional imaging such as ^68^Ga-DOTATATE PET being considered. The updated international consensus guidelines state that all patients with TSC should have a baseline abdominal CT or MRI, with further imaging contingent on the original findings or the development of symptoms (5). It should be noted that in our small case series, one pNET was detected in a patient as young as 13 years of age. In the TOSCA (*TuberOus SClerosis registry to increase disease Awareness*) dataset, 2 patients aged 9 to 14 years were recorded as having a pancreatic tumor (47). We recommend that these patients are discussed at both TSC and NET tumor boards, and their overall care coordinated between the NET and TSC centers.

The clinical decision to remove a pNET surgically in this cohort involves weighing up their malignant and secretory potential with risks of intra- and postoperative complications (64). A recent publication of a pediatric cohort from Boston Children’s Hospital, USA, reported similar findings regarding the management of TSC-associated pNETs, further supporting the role of surveillance (50). Due to a lack of specific guidelines, management decisions for TSC-pNETs should align with recommendations for sporadic pNETs, with consideration of surgical resection for solitary, nonfunctioning, well-differentiated tumors larger than 2 cm and a conservative approach for well-differentiated pNETs smaller than 2 cm. Functioning pNETs should generally be resected where possible, given the higher morbidity associated with hormonal symptoms. Additionally, wherever feasible, a parenchyma-sparing surgical approach should be considered (13, 28). These recommendations align with recently published European clinical practice guidelines and expert recommendations for GEP-NETs in children and adolescents, and ENETS guidelines for the management of GEP-NETs (13, 28).

While there is no specific evidence that TSC-pNET behave in a similar manner to sporadic tumors, some literature suggests a more conservative approach could be taken. In our cohort of 26 patients, 1 patient who had a grade 2 glucagonoma, died due to complications of their metastatic pNET 10 years after detection. One review described 10-year mortality rates of 0% in nonfunctioning TSC-pNETs compared with 11.3% in sporadic pNETs, while another reported no incidence of metastases in 16 cases (19, 26). The location of the tumor, as well as its grading, also plays a significant role when considering surgical resection. Lesions in the body or tail of pancreas can be removed with distal pancreatectomy and possible splenectomy, often laparoscopically or robotically. Conversely, lesions in the head or uncinate process of the pancreas would require a pancreatico-duodenectomy (Whipple procedure), which carries a significant morbidity. Hence, consideration of size, rate of growth and risk of metastases need to be considered prior to embarking on surgery: Evans and colleagues have reported low peri-operative and postoperative complications in a series of 10 TSC-pNET patients (18). The 4 reported cases of metastatic disease in nonfunctioning TSC-pNETs suggest that, in common with sporadic pNETs, they may have more aggressive potential than reported previously, further emphasizing the importance of regular surveillance. Of these 4 patients, one had G1 disease, 2 had G2 disease and 1 had G3 disease, and in alignment with guidelines of sporadic pNET cases (11), tumor grade remains a strong determinant of recurrence risk and prognosis; it is this subset of higher-grade disease patients that would likely require closer radiological surveillance and likely benefit from earlier and more aggressive treatment strategies. As most patients with TSC require lifelong surveillance, effective transition from pediatric to adult specialist services is essential. Structured transition pathways including multidisciplinary coordination of care help maintain continuity of imaging surveillance, endocrine follow-up, and assessment for disease progression while reducing the risk of loss to follow-up during adolescence and early adulthood (53, 65).

Everolimus is particularly attractive in TSC-associated pNETs because it directly targets mTOR signaling, the key molecular pathway dysregulated by loss of TSC1/TSC2 function, providing a biologically rational therapeutic strategy (66, 67). It has proven efficacy in the management of the renal, lung, and brain manifestations of TSC, and in the UK, it is licensed to use in patients with TSC for epilepsy (from age 2), renal angiomyolipoma (adults only), and subependymal giant cell astrocytoma (from age 1) (67, 68). Clinical trials have demonstrated that it has efficacy in advanced sporadic pNETs (57, 69) and there is evidence that it is associated with reduced growth rates in TSC-pNETs (18, 19); mTOR inhibitors were commenced in response to the detection of 4 of the pNETs. The tumor remained stable in one case, while in another, the tumor shrunk from 3.7 to 3 cm (19%) over 4 years (grade 1 disease). The cases suggest a role for mTOR inhibitors in the management of pNETs associated with TSC. Of the other 2 patients started on mTOR inhibitors, one died of a probable unrelated cause, and the other has developed progression of grade 3 metastatic disease. Data regarding metformin exposure were unavailable across published studies and were unavailable for all of patients in our retrospective cohort, precluding assessment of any association with tumor behavior or response to therapy. Although experimental studies suggest metformin may indirectly inhibit mTOR signaling, clinical evidence supporting its role in TSC-associated pNETs remains insufficient (70).

### Limitations

The retrospective nature of our data collection limits our understanding of clinical context. Our cases show heterogeneity, with some TSC-pNETs remaining stable with a surveillance approach, and disease progression in others. We have limited information on factors influencing this. This leaves us vulnerable to information bias and limits our understanding of the clinical context of each of these presentations. Furthermore, in common with all literature describing the behavior of pNETs in TSC, our study is limited by the relatively small sample sizes and low statistical power: 6 of our cases were diagnosed within the last 2 years, which limits the extent to which we can make conclusions regarding long-term outcomes of management.

## Conclusions

Our cases demonstrate variable clinical behavior and outcomes of pNETs in TSC. The majority of pNETs in our cohort are nonfunctioning tumors, although we did identify 2 glucagonomas. We thus speculate that there has been selective reporting of the occurrence of insulinomas in TSC in previous series. While most TSC-pNETs are low grade and indolent, we report the occasional case of postoperative recurrence and metastases, predominantly in the setting of higher tumor grade, and it is this subset of patients that may warrant closer surveillance. Implementation of existing guidelines for the management of TSC-pNET requires further evaluation of how tumor phenotype predicts clinical course and this will likely include further molecular profiling in due course. Until then, there is insufficient evidence base to deviate from the sporadic pNET recommendations. Our limited data support previous evidence that mTOR inhibitors can slow the growth rate of TSC-pNETs. Standardized prospective data collection is needed to establish whether this resembles a viable alternative to surgery. International collaboration and data collection registry for this rare tumor would help standardize data collection and reporting and therefore better inform future clinical guidelines.

## Data Availability

Original data generated and analyzed during this study are included in this published article or in the data repositories listed in References.

## Abbreviations

CT: computed tomography
GEP-NET: gastro-enteropancreatic neuroendocrine tumor
MEN1: multiple endocrine neoplasia type 1
MRI: magnetic resonance imaging
mTOR: mechanistic target of rapamycin
NF1: neurofibromatosis type 1
pNET: pancreatic neuroendocrine tumor
PRISMA: Preferred Reporting Items for Systematic Reviews and Meta-Analyses
TSC: tuberous sclerosis complex
USS: ultrasound scan
VHL: von Hippel-Lindau
